# Dual functional WO_3_/BiVO_4_ heterostructures for efficient photoelectrochemical water splitting and glycerol degradation

**DOI:** 10.1039/d3ra02691d

**Published:** 2023-06-22

**Authors:** Piangjai Peerakiatkhajohn, Jung-Ho Yun, Teera Butburee, Miaoqiang Lyu, Chawalit Takoon, Supphasin Thaweesak

**Affiliations:** a Faculty of Environment and Resource Studies, Mahidol University Nakhon Pathom 73170 Thailand piangjai.pee@mahidol.ac.th; b Department of Environmental Science and Engineering, College of Engineering, Kyung Hee University 1732 Deogyeong-daero, Giheung-gu Yongin-si Gyeonggi-do 17104 Republic of Korea jungho.yun@khu.ac.kr; c National Nanotechnology Center, National Science and Technology Development Agency 111 Thailand Science Park Pathum Thani 12120 Thailand teera.but@nanotec.or.th; d Nanomaterials Centre, School of Chemical Engineering and Australian Institute for Bioengineering and Nanotechnology (AIBN), The University of Queensland St Lucia QLD 4123 Australia m.lyu@uq.edu.au; e Mahidol University Frontier Research Facility (MU-FRF), Mahidol University Nakhon Pathom 73170 Thailand chawalit.tak@mahidol.ac.th; f Department of Chemical Engineering, Faculty of Engineering, Burapha University Chon Buri 20131 Thailand supphasin@eng.buu.ac.th

## Abstract

Dual functional heterojunctions of tungsten oxide and bismuth vanadate (WO_3_/BiVO_4_) photoanodes are developed and their applications in photoelectrochemical (PEC) water splitting and mineralization of glycerol are demonstrated. The thin-film WO_3_/BiVO_4_ photoelectrode was fabricated by a facile hydrothermal method. The morphology, chemical composition, crystalline structure, chemical state, and optical absorption properties of the WO_3_/BiVO_4_ photoelectrodes were characterized systematically. The WO_3_/BiVO_4_ photoelectrode exhibits a good distribution of elements and a well-crystalline monoclinic WO_3_ and monoclinic scheelite BiVO_4_. The light-absorption spectrum of the WO_3_/BiVO_4_ photoelectrodes reveals a broad absorption band in the visible light region with a maximum absorption of around 520 nm. The dual functional WO_3_/BiVO_4_ photoelectrodes achieved a high photocurrent density of 6.85 mA cm^−2^, which is 2.8 times higher than that of the pristine WO_3_ photoelectrode in the presence of a mixture of 0.5 M Na_2_SO_4_ and 0.5 M glycerol electrolyte under AM 1.5 G (100 mW cm^−2^) illumination. The superior PEC performance of the WO_3_/BiVO_4_ photoelectrode was attributed to the synergistic effects of the superior crystal structure, light absorption, and efficient charge separation. Simultaneously, glycerol plays an essential role in increasing the efficiency of hydrogen production by suppressing charge recombination in the water redox reaction. Moreover, the WO_3_/BiVO_4_ photoelectrode shows the total organic carbon (TOC) removal efficiency of glycerol at about 82% at 120 min. Notably, the WO_3_/BiVO_4_ photoelectrode can be a promising photoelectrode for simultaneous hydrogen production and mineralization of glycerol with a simple, economical, and environmentally friendly approach.

## Abstract 2

A dual-functional WO_3_/BiVO_4_ photoanode achieved remarkable photocurrent density, 6.85 mA cm^−2^ at 1.23 V *vs.* RHE and high efficiency for glycerol degradation.

## Graphical abstract

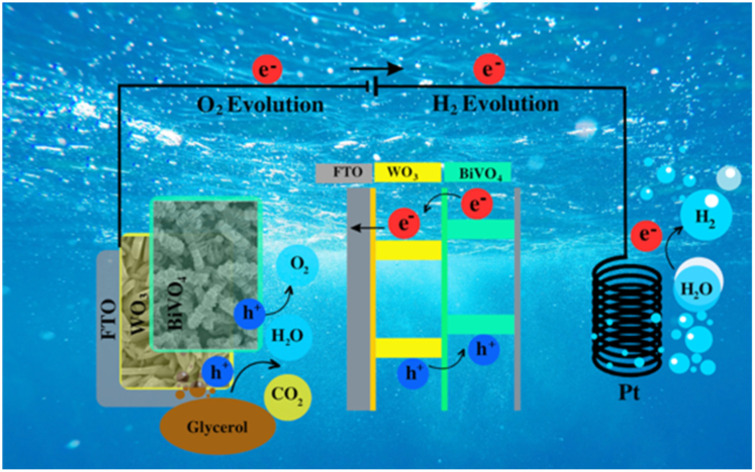

## Introduction

Clean water and clean energy are two urgent global goals to achieve a better and more sustainable future for human society. Biodiesel is a renewable energy that holds various aspects of sustainability.^1^ However, during biodiesel production glycerol by-product is generated in large amounts. Crude glycerol is generated between 60 and 70 wt% from the transesterification process.^2^

Although, glycerol is used widely in various industries such as pharmaceutical, medicine, cosmetics, toiletries, food, and personal care products, the glycerol by-product from biofuel production is still in surplus. Therefore, the surplus crude glycerol has resulted in its price reduction by 60% and has been regarded as a waste stream that required proper disposal.^3,4^ In addition, continuously increasing energy demand and fossil energy exhaustion have driven the energy price to rise rapidly. Furthermore, the combustion of fossil fuels releases carbon dioxide (CO_2_), which is one of the greenhouse gases that primarily causes global warming. To overcome these issues, the next-generation energy source should be sustainable and clean. Hydrogen gas has been considered a high-efficiency, storable, transportable, renewable, and environmentally-friendly energy.^5^

Several engineering strategies have been studies to sustainably produce H_2_ for instance pyrolysis,^6^ thermolysis,^7^ electrolysis,^8–10^ biophotolysis,^11^ and photoelectrolysis.^12^

Photoelectrolysis is a process of water splitting by the integration of solar energy and electric power also known as the photoelectrochemical (PEC) process. The PEC process provides a sustainable way to produce H_2_ due to the most abundant of solar energy. The PEC water splitting has been extensively studied with the aim to improve the efficiency and stability.^13,14^ PEC water splitting from a semiconducting TiO_2_ photoanode under UV illumination was first reported by Fujishima and Honda in 1972.^15^ Semiconductor electrodes play an important role in PEC water splitting. The primary reasons are PEC water splitting process requires three fundamental steps, including light absorption, photoexcited charge separation and transportation, and photoexcited charge reaction.^13^ During the past five decades, earth-abundant and low-cost materials metal oxide semiconductors such as TiO_2_,^16–18^ ZnO,^19^ WO_3_,^20–22^ Fe_2_O_3_,^23,24^ Cu_2_O,^25^ and BiVO_4_ ^26,27^ have been extensively explored as photocatalyst materials in the PEC system. These studies reveal that metal oxide materials have great potential to be promising photoelectrode materials for PEC water splitting. However, using a single semiconductor photocatalyst still holds great challenges for obtaining high PEC performance. For instance, insufficient light absorption,^28^ inefficient charge separation and charge transportation,^29^ and photo-corrosion are the main factors that limit the PEC water splitting performance.^13,14,30^ Fortunately, WO_3_ and BiVO_4_ are two of the most promising photoanode materials, due to their chemically stable, and narrow band gap (WO_3_*E*_g_ = 2.5–2.7 eV, BiVO_4_*E*_g_ = 2.4 eV)^20,26^ Especially, heterostructure WO_3_/BiVO_4_ has been reported to improve charge transfer and alleviate charge recombination.^29,31–33^

Herein, we developed dual-function WO_3_/BiVO_4_ photoelectrodes for both converting energy from renewable energy sources and simultaneously removal of glycerol pollutant. The WO_3_/BiVO_4_ photoelectrode is composed of a superior structure of the monoclinic structure of WO_3_ and monoclinic scheelite structure of BiVO_4_ that provided good electron transport and photocatalytic activity, respectively.

In addition, the constructed type-II heterostructure of WO_3_/BiVO_4_ effectively reduced charge recombination by facilitating electron separation between WO_3_ and BiVO_4_. Furthermore, the oxygen vacancy in the WO_3_/BiVO_4_ further improves the charge separation of WO_3_/BiVO_4_. The photoelectrochemical performance showed that the photocurrent density of the WO_3_/BiVO_4_ photoelectrode was 5.12 mA cm^−2^ at 1.23 V *vs.* RHE under simulated AM 1.5G illumination, which was 2 times higher than that of the pristine WO_3_ photoelectrode in the presence of 0.5 M Na_2_SO_4_ electrolyte. The photocurrent density of the WO_3_/BiVO_4_ photoelectrode was further improved to 6.85 mA cm^−2^ and the TOC reached 80% in the presence of a mixture of 0.5 M Na_2_SO_4_ and 0.5 M glycerol electrolyte. These results highlighted a simple and economical approach to fabricating WO_3_/BiVO_4_ photoelectrodes that exhibit a considerable performance for dual PEC water splitting and contaminant degradation.

## Results and discussion

### Characterization of WO_3_ and WO_3_/BiVO_4_ photoelectrodes

**Fig. 1:**
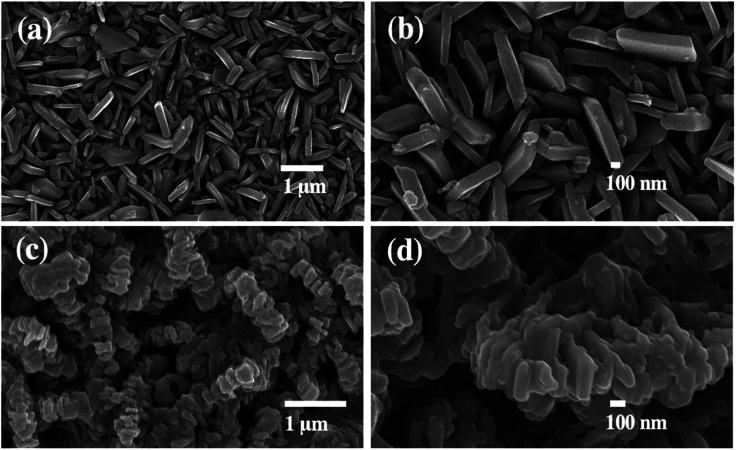
SEM images of (a and b) WO_3_ and (c and d) WO_3_/BiVO_4_ photoelectrodes.

The morphology of the nanostructured photoelectrodes was examined by SEM as shown in Fig. 1(a–d). Fig. 1(a) shows that the FTO substrate is densely and uniformly covered by vertically-aligned two-dimensional (2D) WO_3_ nanoplates. Fig. 1(b) is the higher magnification SEM image showing WO_3_ 2D nanoplates with smooth surface and the thickness estimated to be ∼100 nm. Fig. 1(c) and (d) show the WO_3_ nanoparticles which are deposited by BiVO_4_, which is so called WO_3_/BiVO_4_ thereafter. Obviously, WO_3_/BiVO_4_ exhibits aligned structure, rougher surface, and much thicker than that of the bare WO_3_, indicating successful deposition of BiVO_4_.

**Fig. 2:**
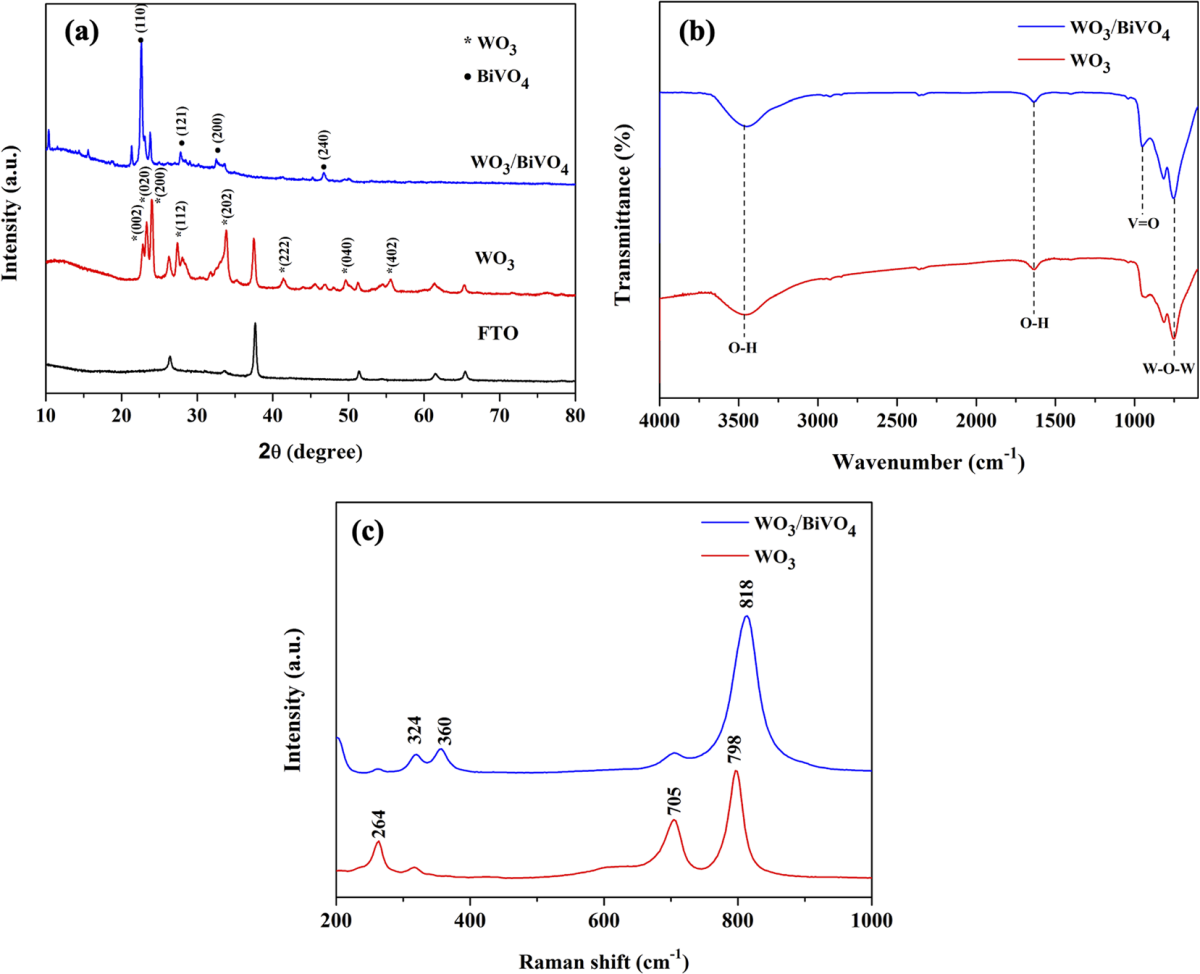
(a) XRD patterns (b) FTIR spectra and (c) Raman spectra of WO_3_ and WO_3_/BiVO_4_ photoelectrodes.

Fig. 2(a) shows X-ray diffraction (XRD) patterns of WO_3_ and WO_3_/BiVO_4_ photoelectrodes. The XRD patterns of WO_3_ were observed at 23.1°, 23.6°, 24.3°, 28.9°, 34.1°, 41.9°, 48.2°, and 55.4°, corresponding to the (002), (020), (200), (112), (202), (222), (040), and (402) planes, respectively. The diffraction peaks can be well indexed to the monoclinic crystal phase of WO_3_ (JCPDS No. 43-1035).^32^ After depositing BiVO_4_ on WO_3_, the XRD patterns of the WO_3_/BiVO_4_ shows diffraction peaks at 18.6°, 28.8°, 34.5°, and 46.7°, corresponding to the (110), (121), (200), (240) planes of the monoclinic scheelite structure of BiVO_4_ (JCPDS No. 14-0688), respectively.^34^ In addition, no obvious diffraction peaks of WO_3_ in the heterostructure WO_3_/BiVO_4_, and no other peaks are observed, suggesting that relatively thick layer of BiVO_4_ deposited on the WO_3_ surface. In addition, both monoclinic structure of WO_3_ and monoclinic scheelite structure of BiVO_4_ have been reported to have superior properties, where the monoclinic of WO_3_ phase was reported to have faster electron transport than orthorhombic phase.^35^ Moreover, the monoclinic scheelite structure of BiVO_4_ was reported as the most photocatalytic active phase among the other two crystal structures, tetragonal scheelite structure, and tetragonal zircon structure.^36^ The WO_3_ and WO_3_/BiVO_4_ photoelectrodes were analyzed by FTIR to investigate the presence of functional groups, as shown in Fig. 2(b). The peaks at 3500 and 1635 cm^−1^ can be ascribed to the stretching vibration and the bending mode of the hydroxyl group (–OH), respectively. The hydroxyl group found was due to the atmospheric humidity adsorbed onto the surface.^31^ In addition, the hydroxyl group provides a beneficial effect for trapping charge carriers to create reactive hydroxyl radical (OH˙) that effectively oxidizes organic molecules.^37^ The crystal structure of WO_3_ and WO_3_/BiVO_4_ was further investigated by Raman spectroscopy. Fig. 2(c) the Raman spectrum of WO_3_ showing three distinctive peaks at 264, 705, and 798 cm^−1^, which can be ascribed to the O–W–O bending vibration and the W–O–W stretching vibration.^31,38^ After BiVO_4_ was deposited on WO_3_, the Raman spectrum of WO_3_/BiVO_4_ reveals new peaks at 324 and 360 cm^−1^, indicating the asymmetric and symmetric deformation modes of the VO_4_^3−^, respectively.^31^ These results indicate that WO_3_/BiVO_4_ is successfully fabricated. In addition, it is obvious that the most intense peak of the WO_3_ and WO_3_/BiVO_4_ shifts from 798 to 813 cm^−1^, suggesting non-stoichiometric of WO_3_/BiVO_4_ due to oxygen vacancies.

**Fig. 3:**
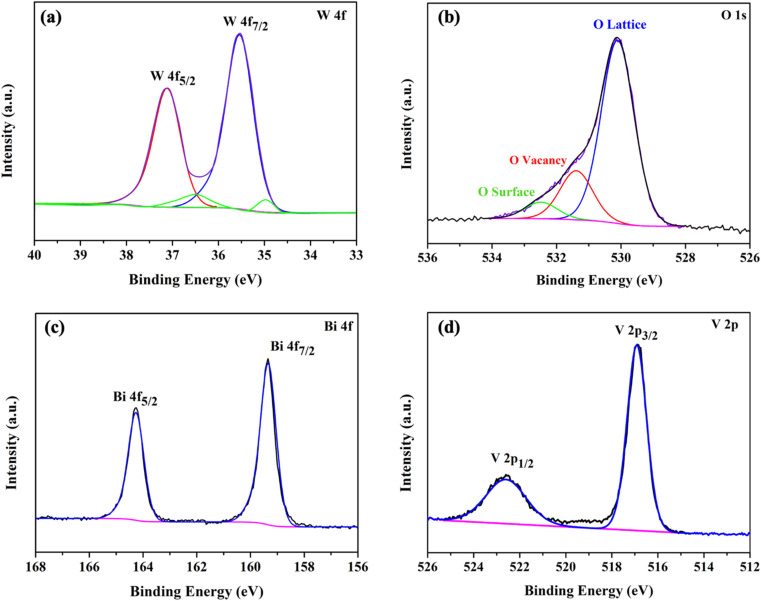
XPS spectra of (a) W 4f (b) O 1s (c) Bi 4f and (d) V 2p of WO_3_/BiVO_4_ photoelectrodes.

To confirm the presence of oxygen vacancies, the chemical state of the WO_3_/BiVO_4_ photoelectrodes was analyzed by XPS. Fig. 3(a–d) show high resolution spectra for the W 4f, O 1s, Bi 4f, and V 2p, respectively. As shown in Fig. 3(a), the W 4f spectra reveals four curves after convolution using Gaussian distribution. The two intense peaks at 35.3 and 37.4 eV can be ascribed to W 4f_7/2_ and W 4f_5/2_ of W^6+^ state. The two low-intensity peaks at 34.6 and 36.7 eV corresponding to W 4f_7/2_ and W 4f_5/2_ of W^5+^ state, indicating the presence of oxygen vacancies defects.^39^Fig. 3(b) shows O 1s spectra with a broad shoulder to the higher binding energy side, indicating the existence of several oxygen species. The intense peak at 530.1 eV corresponds to the lattice oxygen in metal oxides (O Lattice), while the other two oxygen peaks at 531.4 and 532.5 eV can be assigned to the surface defects (O vacancy), and the surface absorbed oxygen (O surface), respectively.^31,40^Fig. 3(c) reveals two typical peaks at 159.4 and 164.3 eV corresponding to Bi 4f_7/2_ and Bi 4f_5/2_ of Bi^3+^ state.^38^ Furthermore, Fig. 3(d) shows V 2p spectra with two peak located at 516.9 and 522.7 eV, which can be assigned to V 2p_3/2_ and V 2p_1/2_ of V^5+^ state, respectively.^31^

**Fig. 4:**
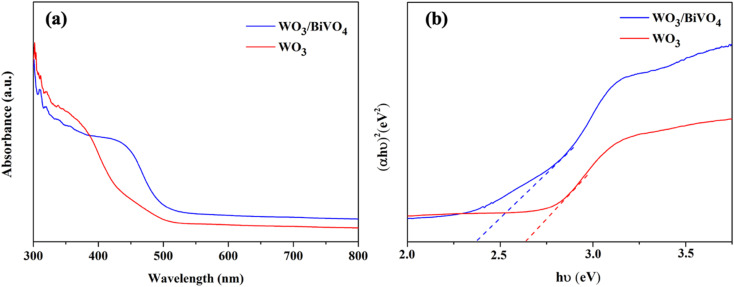
(a) UV-Vis spectra and (b) energy bandgap of WO_3_ and WO_3_/BiVO_4_ photoelectrodes.

**Fig. 5:**
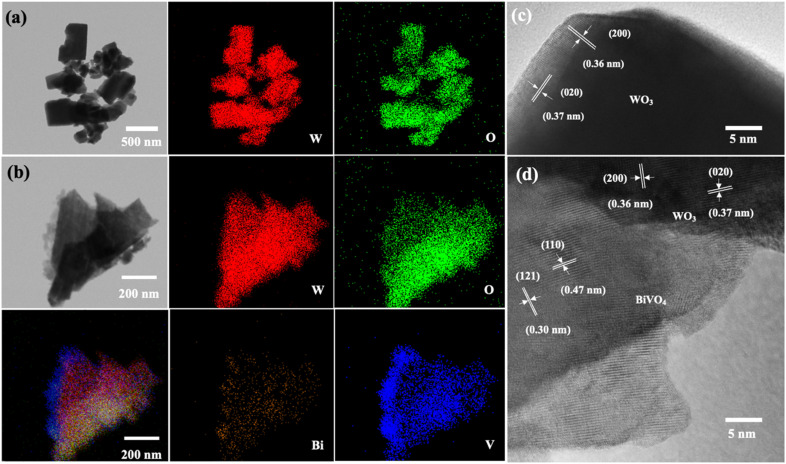
TEM images and the corresponding EDS elemental mappings of (a) representative bare WO_3_ (b) WO_3_/BiVO_4_ photoelectrodes and HRTEM of (c) WO_3_ (d) WO_3_/BiVO_4_ photoelectrodes.

The light absorption spectra of the photoelectrodes were analyzed using the UV-Vis DRS technique to reveal the light-harvesting property as shown in Fig. 4(a). The absorption edge of the WO_3_ nanoplates is around 470 nm, while the absorption range was extended to around 520 nm for WO_3_/BiVO_4_. The band gap of the WO_3_ and WO_3_/BiVO_4_ are 2.64 and 2.38 eV, respectively as shown in Fig. 4(b). The WO_3_/BiVO_4_ samples showed good absorption in the visible light region, which can facilitate the enhancement of PEC properties under visible light irradiation.^33,41^Fig. 5(a) and (b) show TEM images and the corresponding EDS elemental mapping of WO_3_ and WO_3_/BiVO_4_ photoelectrodes. As shown in Fig. 5(a), W and O elements are uniformly distributed on the nanoplates without other impurities.

The weight percentage of W and O elements were estimated at 80.7 wt% and 19.3 wt%, respectively. Fig. 5(b) shows the elemental mapping of WO_3_/BiVO_4_ photoelectrode. The result shows that W and O are uniformly distributed throughout the whole particle, while Bi and V also presented in the elemental maps, indicating the existence of BiVO_4_ in the WO_3_/BiVO_4_ composite. The weight percentages of W, O, Bi, and V elements were estimated at 59.3 wt%, 17.5 wt%, 19.3 wt%, and 3.8 wt%, respectively. Furthermore, the high-resolution transmission electron microscope (HRTEM) images of the selected WO_3_ and WO_3_/BiVO_4_ nanoplates were conducted as shown in Fig. 5(c) and (d).

Fig. 5(c) shows the measured lattice fringes with interface spacings of 0.36 nm and 0.37 nm which corresponded to the (200) and (020) crystallographic planes of monoclinic WO_3_ crystal phase, respectively, suggesting that WO_3_ nanoplates have highly crystallized monoclinic structure. Fig. 5(d) shows the HRTEM of the composite WO_3_/BiVO_4._ It reveals that the lattice fringe with interface spacings of 0.36 nm and 0.37 nm, which belong to the WO_3_ monoclinic structure and additional lattice fringe with interface spacings of 0.30 and 0.47 nm that can be ascribed to the (121) and (110) crystal planes of BiVO_4_, respectively. These HRTEM results are in agreement with the XRD and confirm that WO_3_ nanoplates closely contact with BiVO_4_.

### Photoelectrochemical performance

**Fig. 6:**
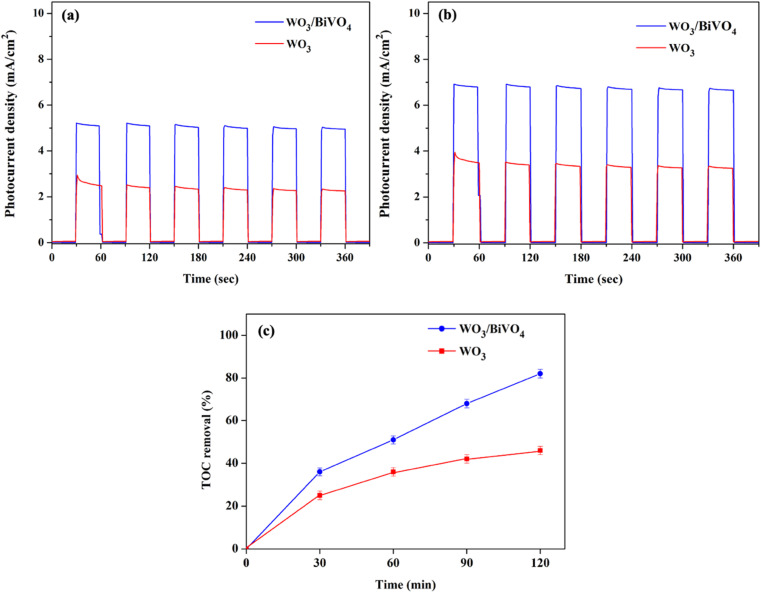
Transient photocurrent response (*I*–*t*) of WO_3_ and WO_3_/BiVO_4_ photoelectrodes in the presence of (a) 0.5 M Na_2_SO_4_ (b) 0.5 M Na_2_SO_4_ + 0.5 M glycerol at 1.23 V *vs.* RHE under simulated AM 1.5 G illumination and (c) TOC removal efficiency of glycerol.

The PEC performance of WO_3_ and WO_3_/BiVO_4_ photoelectrodes was evaluated by measuring the transient photocurrent response (*I*–*t* curve) in the presence of 0.5 M Na_2_SO_4_ and an equal concentration of Na_2_SO_4_ and glycerol at 0.5 M electrolyte at the applied potential of 1.23 V (*vs.* RHE) under simulated AM 1.5 G illumination as shown in Fig. 6(a and b). Fig. 6(a) shows the photocurrent density of WO_3_ and WO_3_/BiVO_4_ photoelectrodes in presence of 0.5 M Na_2_SO_4_ electrolyte. The pristine WO_3_ photoelectrode photocurrent density is 2.40 mA cm^−2^ which is comparable to other reported PEC systems of WO_3_ photoelectrode.^29,31,32^ Interestingly, the photocurrent density of the WO_3_/BiVO_4_ photoelectrode increases to 5.12 mA cm^−2^, approximately 2 times higher than that of the pristine WO_3_ photoelectrode. The improved photocurrent density of WO_3_/BiVO_4_ photoelectrode could be attributed to a synergistic effect of the crystal structure, light absorption, and charge separation. First, the monoclinic structure of WO_3_ and the monoclinic scheelite structure of BiVO_4_ provide fast electron transport and superior photocatalytic activity, respectively. Second, the light absorption of WO_3_/BiVO_4_ samples as shown in Fig. 4(a) that the absorption range was extended to around 520 nm compared to WO_3_ nanoplates that have the main absorption edge around 470 nm. Thus, good absorption in the visible light region can facilitate the enhancement of PEC properties under visible light irradiation.^31^ Third, the WO_3_/BiVO_4_ forms a type-II heterostructure which effectively reduces charge recombination by facilitating electron separation between WO_3_ and BiVO_4_.^32,38^ In addition, the oxygen vacancy presence in WO_3_/BiVO_4_ from the XPS analysis as shown in Fig. 3(b) can increase the driving force for charge separation of WO_3_/BiVO_4_.^31,38^ To further improve the performance and additional functionality of PEC for H_2_ production and waste degradation, glycerol was added to the NaSO_4_ electrolyte to generate a bifunctional PEC system. It has been reported that small organic molecules such as glycerol waste can act as a sacrificial agent and electron donor, promoting the photocatalytic water splitting performance.^42,43^ As shown in Fig. 6(b), the photocurrent density of WO_3_ and WO_3_/BiVO_4_ photoelectrodes in presence of a mixture of 0.5 M Na_2_SO_4_ and 0.5 M glycerol electrolyte is substantially increased. The photocurrent response of bare WO_3_ photoelectrode is 3.41 mA cm^−2^ and increases to 6.85 mA cm^−2^ for WO_3_/BiVO_4_ photoelectrodes. Interestingly, the photocurrent response of WO_3_/BiVO_4_ photoelectrodes in the presence of Na_2_SO_4_ and glycerol in an electrolyte compared to that of WO_3_ photoelectrode in the presence of only NaSO_4_ was increased by 2.8 times. The superior performance of WO_3_/BiVO_4_ photoelectrodes is due to the above-mentioned reasons and possibly due to the hydroxy group, which provides trapping for photoexcited holes and creates reactive hydroxyl radical (OH˙) that effectively oxidize organic molecule,^37^ from FTIR analysis as shown in Fig. 2(b). In addition, Fig. 6(c) shows the TOC removal by WO_3_ and WO_3_/BiVO_4_ photoelectrodes. This can be seen that the TOC removal of WO_3_/BiVO_4_ photoelectrodes substantially increased and reached 82%, while WO_3_ photoelectrodes gradually increased and reached 40%. Thus, the WO_3_/BiVO_4_ photoelectrodes provide efficient charge transfer and separation, which in turn contribute to both effective PEC water splitting and contaminant degradation.

**Fig. 7:**
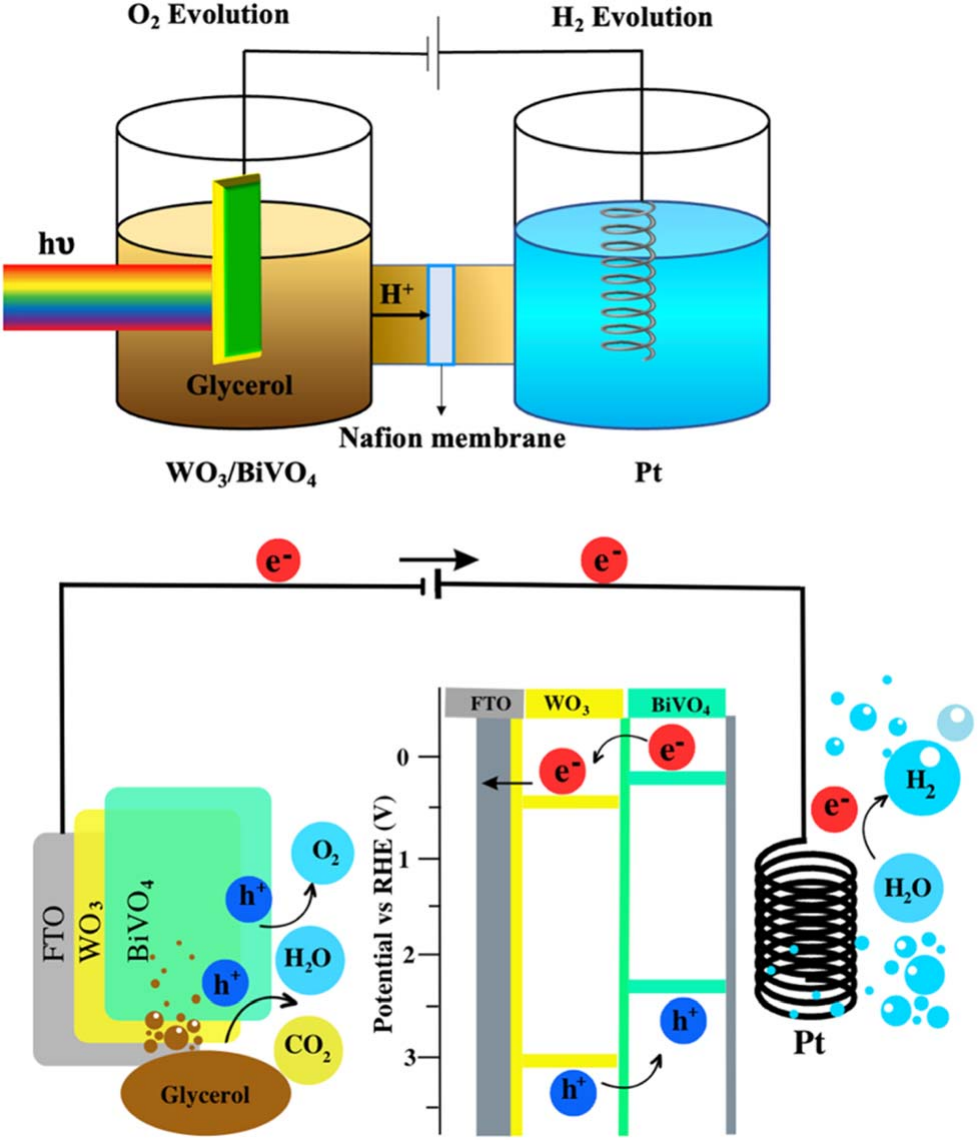
Schematic visualization of the bifunctional PEC system (hydrogen generation and glycerol degradation) using H-cell type PEC reactor.

Based on the above discussions, the mechanism of the dual functional PEC system of photoelectrode is illustrated in Fig. 7, the conduction band (CB) of BiVO_4_ is close to the hydrogen-reduction potential and the photo-excited electrons can thermodynamically transfer from the high CB energy level of BiVO_4_ to the more positive CB of WO_3_. Holes in the valence band (VB) of WO_3_ can move spontaneously to the VB of BiVO_4_ for water oxidation. These bandgap differences between WO_3_ and BiVO_4_ also enhance the charge separation and reduce the bulk's charge recombination rate. As a result, the WO_3_/BiVO_4_ photoelectrodes exhibit much better PEC performance than that of the bare WO_3_ photoelectrode.

## Experimental section

### Synthesis of WO_3_ and WO_3_/BiVO_4_ photoelectrodes

**Fig. 8:**
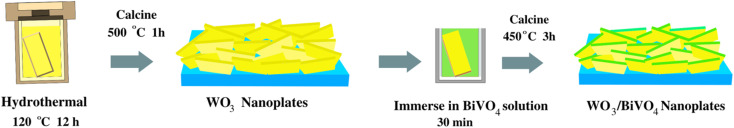
Schematic figure summarizing the fabrication processes for synthesizing WO_3_/BiVO_4_ photoelectrodes.

Fig. 8 shows the preparation of WO_3_ and WO_3_/BiVO_4_ photoanodes by a hydrothermal method. Firstly, 0.1 g of sodium tungstate dihydrate (Na_2_WO_4_·2H_2_O) was dissolved in 10 mL of Milli-Q water. Then, 3 mL of 2 M of hydrochloric acid (HCl) was added dropwise in the above solution under constant stirring at room temperature for 15 min. Subsequently, 0.2 g of citric acid (C_6_H_8_O_7_) and 15 mL of Milli-Q water were added into the mixture with continual stirring for 20 min. Afterward, 30 mL of the prepared precursor solution was then transferred to a 50 mL Teflon-lined stainless-steel autoclave. Before immersing FTO glass into the autoclave, FTO glass substrate was cleaned by ultrasonic treatment using acetone, ethanol, and isopropanol (each for 15 min), followed by drying in a nitrogen stream. After the FTO glass was immersed in an autoclave with the FTO side leaned down against the wall, the autoclave was sealed and kept at 120 °C for 8 h. After the hydrothermal process, the samples were rinsed with Milli-Q water, and dried in a nitrogen stream. Finally, WO_3_ photoelectrodes were obtained after annealing in air at 500 °C for 1 h. For WO_3_/BiVO_4_ photoelectrodes, the precursor of BiVO_4_ was prepared by dissolving 1.4 g of bismuth(iii) nitrate pentahydrate (Bi(NO_3_)_3_·5H_2_O), 0.8 g of vanadium acetylacetonate (C_10_H_14_O_5_V), 1 mL of acetic (CH_3_COOH), and 19 mL of acetylacetone (C_5_H_8_O_2_) with sonication until the solution's color changed to dark green. Then, the prepared WO_3_ photoelectrode was immersed into the BiVO_4_ precursor solution for 30 min. Afterward, the above sample was annealed in air at 450 °C for 3 h to obtain the WO_3_/BiVO_4_ photoelectrodes.

### Characterization

The surface morphology of the as-prepared photoelectrodes were examined using a field emission scanning electron microscope (FE-SEM, JSM-7610FPlus, JEOL, Tokyo, Japan). Transmission electron microscope equipped with an energy-dispersive X-ray spectroscope (TEM/EDX) and high-resolution TEM (HRTEM) analyses were conducted by JEOL2100 Plus, operated at 200 keV. The crystalline phases of the photoanodes were characterized by X-ray diffraction (XRD; Bruker, D8 Discover, Germany) using the Cu Kα radiation in a 2*θ* range of 20°–80°. The functional group of the photoelectrodes were analyzed by FTIR spectroscopy and recorded over a region of 4000–650 cm^−1^ (Nicolet 6700, Thermo Scientific, USA). Raman spectra were recorded over a spectral range of 200–1000 cm ^−1^ and collected on a Horiba XploRA PLUS instrument, Japan. The elemental states were analyzed by X-ray photon spectroscopy (XPS, Kratos AXIS Ultra DLD). The light absorption spectra were investigated by a UV-Vis spectrophotometer (JASCO V-630).

### Photoelectrochemical measurement

The dual functional PEC measurements were performed in an H-cell type PEC cell with a quartz window and tested on a CHI 660D electrochemical workstation. The prepared photoelectrodes, a Pt wire (1 mm diameter), and a Ag/AgCl electrode served as the working electrode, counter electrode, and reference electrode, respectively. The illumination area was set by an aperture diameter of 1 cm. A xenon lamp (100 W, Newport LCS-100) was used to simulate sunlight and the photocurrent densities were measured under solar AM 1.5 G. The PEC behaviour of the electrodes was characterized with degradation of 100 mL of a mixture of 0.5 M glycerol and 0.5 M Na_2_SO_4_ solution at room temperature. All solutions were prepared from Milli-Q water. The solutions were purged with nitrogen gas for 30 min prior to PEC measurement. To evaluate the degradation of glycerol, 5 mL of the treated solution was sampling every 30 min and analyzed by a Shimadzu TOC-V CPN Total Organic Carbon Analyzer. A 150 W Xe lamp light source with the intensity of the simulated 1-Sun solar illumination condition (AM 1.5, 100 mW cm^−2^) illuminated at the immersed photoelectrode in the solution. Potentials *versus* RHE were calculated using the Nernst equation *E*_RHE_ = *E*_Ag/AgCl_ + 0.0591(pH) + 0.1976 V.

## Conclusion

In summary, a simple hydrothermal technique is developed for fabricating the heterojunction WO_3_/BiVO_4_ photoanodes. The WO_3_/BiVO_4_ photoanodes take advantage of both the superior electron transport of the monoclinic structure of WO_3_ and the good photocatalytic activity of the monoclinic scheelite structure of BiVO_4_. The photocatalytic activity of the pristine WO_3_ and WO_3_/BiVO_4_ photoanodes are 2.40 mA cm^−2^ and 5.12 mA cm^−2^ in the presence of 0.5 M Na_2_SO_4_ electrolyte, respectively. The improved photocurrent density of WO_3_/BiVO_4_ photoelectrode could be attributed to a synergistic effect of the superior crystal structure, type-II heterostructure, and the presence of oxygen vacancies. Furthermore, the photocurrent density of WO_3_/BiVO_4_ photoelectrodes was improved to 6.85 mA cm^−2^ and the TOC removal efficiency reached about 82%, in the presence of a mixture of 0.5 M Na_2_SO_4_ and 0.5 M glycerol electrolyte. The photocurrent density of the WO_3_/BiVO_4_ photoelectrodes is about 2.8 times higher than that of the pristine WO_3_ in the presence of 0.5 M Na_2_SO_4_ electrolyte. The considerable enhancement is due to the afore-mentioned synergistic effects and the hydroxy group that provides trapping for photoexcited holes and creates reactive hydroxyl radicals (OH˙) that effectively oxidizes organic molecules.

## Author contributions

“Conceptualization, P. P., J.-H. Y., T. B., and S. T.; methodology, P. P., and S. T.; validation, P. P., J.-H. Y., T. B., M. L., C. T., and S. T.; formal analysis, P. P., J.-H. Y., T. B., M. L., C. T., and S. T.; investigation, P. P., J.-H. Y., T. B., M. L., and S. T.; resources, P. P., T. B., C. T., and S. T.; visualization, P. P.; writing—original draft preparation, P. P., and S. T.; writing—review and editing, P. P., J.-H. Y., T. B., M. L., C. T., and S. T.; and supervision, S. T. All authors have read and agreed to the published version of the manuscript.”

## Conflicts of interest

There are no conflicts to declare.

## Supplementary Material
